# Impact of intraoperative tidal volume on surgical parameters during flexible ureteroscopy

**DOI:** 10.1007/s10103-026-04894-5

**Published:** 2026-06-03

**Authors:** Ersin Gokmen, Seniyye Ulgen Zengin, Turker Altuntas, Ceyhun Ozdemiroglu, Bahadir Kagan Kalyoncu, Dogancan Dorucu, Gunal Ozgur, Meliha Orhon Ergun, Tarik Emre Sener

**Affiliations:** 1 Department of Urology, Marmara Üniversitesi Eğitim ve Araştırma Hastanesi, Istanbul, Turkey; 2 Department of Anesthesiology, Marmara University, Istanbul, Turkey; 3 Department of Urology, Marmara University, Istanbul, Turkey

**Keywords:** Flexible ureteroscopy, Laser efficiency, Tidal volume, Urolithiasis

## Abstract

To investigate the impact of intraoperative tidal volume on surgical efficiency and outcomes in patients undergoing flexible ureteroscopy (fURS). This prospective cohort study included adult patients with unilateral, solitary renal stones located in the upper or middle calyx or in the renal pelvis. Patients were divided into two groups based on intraoperative tidal volume settings. Demographic factors, stone features, operative metrics, laser efficiency, complication rates, and hospital stay were compared. A total of 70 patients were included (median age: 49 years). Forty-two patients (60%) had a tidal volume ≥ 500 mL (Group 1) and 28 (40%) had a tidal volume < 500 mL (Group 2). Baseline characteristics, including age, ASA score, BMI, comorbidities, stone size, volume, density, laterality, and location, were comparable between groups, except for a higher proportion of males in Group 1 (*p* < 0.001). Operative time and lasing time did not differ significantly between groups. Total lasing energy was higher in Group 1 (*p* = 0.046), while laser efficiency was significantly greater in Group 2 (*p* = 0.031). Intraoperative ventilatory parameters and postoperative outcomes, including stone-free rates, infection rates, and hospital stay, were similar between groups. Within physiological limits, lower tidal volumes were associated with higher laser efficiency, an exploratory metric, and lower total laser energy, without adversely affecting operative time, stone-free rates, or perioperative safety. Low tidal volume ventilation was associated with differences in laser efficiency parameters during fURS; however, these findings should be interpreted as exploratory and hypothesis-generating.

## Introduction

Among urinary tract diseases, urolithiasis ranks as the third most common condition. Its prevalence is steadily increasing in both men and women, and it is considered a significant global health problem [1]. Epidemiological studies have reported a prevalence ranging from 2% to 20% [2].

With the advancement of technology on flexible ureteroscopes and lasers, endourological interventions, particularly flexible ureteroscopy (fURS) have gained popularity worldwide [3]. In line with the latest European Association of Urology (EAU) recommendations, flexible ureteroscopy is considered a first-line treatment option for renal stones smaller than 2 cm and proximal ureteric stones [4].

Additionally, prolonged operative time increases the risk of life-threatening complications such as infection and bleeding [5, 6]. Therefore, operative time management is crucial to protect patients from these adverse effects. Surgeon experience, stone location and mobility, and kidney movement caused by patient ventilation can all influence the duration of the procedure [7].

Flexible ureteroscopy is most commonly performed under general anaesthesia. During general anaesthesia, patients are maintained within physiologic tidal volume ranges by the anaesthesia team. The physiological tidal volume range is generally 6–10 mL/kg, with a respiratory rate of 8–10 breaths per minute [8].

During fURS, respiratory-induced kidney motion may affect surgical precision by altering stone position and stability. Variations in intraoperative tidal volume can influence the amplitude of renal movement, potentially impacting laser targeting and fragmentation efficiency. Lower tidal volumes may reduce respiratory-related kidney movement, thereby allowing more stable laser–stone interaction and improving procedural efficiency. However, the clinical implications of tidal volume modulation during fURS remain insufficiently defined.

The effect of ventilatory tidal volume on RIRS has not been adequately investigated in previous studies. The available literature on this topic is limited [9, 10]. We hypothesized that the use of lower tidal volume ventilation during fURS could improve surgical conditions by reducing kidney motion and intrarenal pressure fluctuations, thereby enhancing surgical efficiency and perioperative outcomes. Therefore, we aimed to evaluate the impact of intraoperative tidal volume settings on surgical efficiency, stone-free rates, and perioperative outcomes in patients undergoing fURS.

## Materials and methods

This study was a single-centre, prospective cohort study conducted in our tertiary centre. The study was approved by the Institutional Review Board (IRB) where the study took place with the protocol number of Local Ethics Committee 06.2024.729. This study was prospectively registered in the ClinicalTrials.gov (NCT07304297). Informed consent was obtained by all patients when they were enrolled.

Between October 2025 and December 2025, patients who underwent fURS surgery in our clinic, with the surgical indication determined according to the EAU Guidelines, were included in the study [4]. Patients over 18 years of age with unilateral, single stones located in the upper or middle calyx or in the renal pelvis were included in the study. Patients with significant pulmonary disease, pregnancy, a history of abdominal, retroperitoneal, or thoracic surgery, kidney anomalies, or stones located in the lower calyx were excluded.

Patients were prospectively enrolled, and intraoperative ventilation strategies, including tidal volume selection, were determined by the attending anaesthesiologist according to routine clinical practice and individual patient characteristics rather than a predefined study protocol. Deep neuromuscular blockade is applied in all patients using train-of-four monitoring. Standard intraoperative monitoring was used to ensure adequate oxygenation and ventilation throughout the procedure. Intraoperative ventilation was performed using a lung-protective strategy with low tidal volumes. Respiratory rate and inspired oxygen fraction (FiO₂) were adjusted when necessary to maintain normocapnia and adequate oxygenation. Patients were continuously monitored using pulse oximetry and capnography. In addition, oxygenation was supported by oxygen reserve index (ORi) monitoring, which allows early detection of changes in oxygen reserve and helps to avoid unnecessary increases in FiO₂. FiO₂ was maintained at standard intraoperative levels and was only increased when clinically indicated. No protocol-driven increase in respiratory rate or FiO₂ was required in either group, as gas exchange parameters remained within normal physiological ranges throughout the procedures.

Patients were evaluated preoperatively using non-contrast abdominal computed tomography (CT) to determine the surgical indication. The following data were recorded: patients’ demographic characteristics, stone properties, operation durations, fluoroscopic imaging performed during surgery, total laser energy and duration used, laser efficiency [11], tidal volumes at 15-minute intervals, peak airway pressure, positive end-expiratory pressure (PEEP), respiratory rate, applied ventilation mode, oxygen saturation, end-tidal carbon dioxide (ETCO₂), and postoperative outcomes including stone-free status, infection, and hospital stay duration.

All patients were monitored according to the standard monitoring recommended by the ASA, including ETCO₂ monitoring [10]. Train-of-four (TOF) monitoring was performed in all patients, and deep neuromuscular blockade was applied. TOF monitoring is the most commonly used peripheral nerve stimulation method to assess neuromuscular blockade, consisting of four consecutive electrical stimuli and evaluation of the evoked muscle responses [12].

An experienced endourologist, who had performed over 500 fURS procedures, conducted all operations. The operations were regularly carried out in the standard lithotomy position using a 7.5 Fr Red Pine Disposable ureteroscope advanced through an 11/13 Fr, 36-cm access sheath, which was placed over a 0.035’’ sensor guidewire while leaving a similar guidewire as a safety wire. Laser lithotripsy was performed in dusting mode using a Quanta 60 W Cyber Ho Holmium: YAG laser at settings of 0.8 J and 15 Hz with a 270-µm laser fiber. Fragmentation or basketing techniques were not routinely employed. Total laser energy delivered (J) was recorded from the laser device, representing the cumulative energy delivered during the procedure, calculated as the product of pulse energy, frequency, and activation time. Routine ureteral stenting was performed in all patients at the end of surgery.

Postoperative infection was diagnosed based on parameters such as fever and elevated acute phase reactants during follow-up. All patients were followed for 1 month postoperatively. Postoperative stone-free status was evaluated at 1 month after surgery using abdominal X-ray imaging. Stone-free status was defined as the absence of residual fragments or the presence of clinically insignificant residual fragments (< 4 mm) [4]. In accordance with the recommendations of the EAU Guidelines, non-contrast abdominal CT was not routinely performed for stone-free assessment [4].

Stone volume was calculated using 3D Doctor software (ABLE Software Corp., Lexington, MA, USA). Axial, sagittal, and coronal images obtained from patients’ non-contrast CT scans were uploaded to the software, and stone volumes were calculated automatically. The laser efficiency parameter was derived by dividing stone volume by the total duration of laser activation (mm³/min) [11].

The primary outcomes were stone-free rate and operative parameters. Laser efficiency was calculated as stone volume divided by total lasing time and was evaluated as an exploratory procedural performance metric rather than a clinical endpoint.

Based on tidal volumes recorded during anaesthesia follow-up, and referencing similar study in the literature, a threshold value of 500 mL was used to classify patients into two groups: a standard-volume group (Group 1) and a low-volume group (Group 2) [9].

First, the demographic data and stone characteristics of these groups were compared. Subsequently, operative times, surgical efficiency (stone volume/operative time ratio), laser times and energies, stone-free rates, infection rates, and differences in length of hospital stay were analysed.

### Statistical analysis

Sample size and power calculations were conducted with the G*Power statistical software. Using a point-biserial correlation framework, the sample size was calculated assuming an effect size of 0.3, a type I error rate of 0.05, and 80% power. According to these parameters, a total of 64 patients were required for the study.

Statistical analyses were performed using IBM SPSS (Statistical Package for the Social Sciences) version 25.0. The Kolmogorov-Smirnov test was used for normality analysis. Non-parametric tests were used due to the non-normal distribution. Nominal data were presented as frequency and percentage values. Continuous variables are reported as median (IQR), whereas categorical variables were assessed using the chi-square test. Statistical significance was defined as a p value less than 0.05.

## Results

In total, 70 patients were enrolled in the study. Of these patients, 45 (64.3%) were male and 25 (35.7%) were female. The median age of the included patients was 49 years (39–54). The median stone diameter was 13 mm (5–36). Among the patients in the study, 42 (60%) had a tidal volume of ≥ 500 mL during the procedure, while 28 (40%) had a tidal volume of < 500 mL.

The median age was comparable between the two groups (48 vs. 51 years, *p* = 0.395). Gender distribution differed significantly between groups, with a higher proportion of male patients in Group 1 (83.3%) compared to Group 2 (35.7%) (*p* < 0.001). The two groups were similar with respect to ASA score, body mass index and comorbidities, including diabetes mellitus, hypertension, and coronary artery disease (all *p* > 0.05). Stone laterality and stone location distribution were comparable between the groups (*p* = 0.770 and *p* = 0.525, respectively). There were no significant differences in stone diameter, stone volume, or stone density between Group 1 and Group 2 (Table 1).

**Table 1:** Comparison of demographic data and stone characteristics

	Group 1 (*n*:42)Standard Tidal Volume	Group 2 (*n*:28)Low Tidal Volume	*P* value
Age (years)	48 (34.5–54)	51 (41.75-57)	0.395
Gender (Male/Female)	35/7 (83.3/16.7)	10/18 (35.7/64.3)	**< 0.001**
ASA Score	2 (1–2)	2 (1.25-2)	0.855
BMI (kg/m^2^)	27.77 (24.56–30.35)	26.01 (23.83–31.02)	0.396
Diabetes Mellitus	11 (26.2)	6 (21.4)	0.649
Hypertension	9 (21.4)	5 (17.9)	0.714
Coronary Artery Disease	9 (21.4)	3 (10.7)	0.202
Stone Side (Right/Left)	21/21 (50/50)	13/15 (46.4/53.6)	0.77
Location of Stones			
Ureter	16 (38.1)	6 (21.4)	0.525
Renal Pelvis	18 (35.7)	12 (42.9)	
Upper Calyx	3 (7.1)	3 (10.7)	
Middle Calyx	8 (19)	7 (25)	
Stone Diameter (mm)	15 (10-19.5)	12 (9.75-16)	0.089
Stone Volume (mm^3^)	1458.75 (543.5-3404.34)	904.78 (495.45-2144.66)	0.217
Stone Density (Hounsfield Unit)	1177 (770–1336)	1214 (971–1337)	0.585

All continuous variables are presented as median (interquartile range, IQR), and categorical variables are presented as numbers and percentages

*ASA* American Society of Anaesthesiologists, *BMI* Body Mass Index

Perioperative parameters are summarized in Table 2. Median operative time did not differ significantly between the groups (91 vs. 93 min, *p* = 0.572). Lasing time showed a trend toward being longer in Group 1; however, this difference did not reach statistical significance (*p* = 0.056). Total lasing energy was significantly higher in Group 1 compared to Group 2 (27.9 vs. 19.08 J, *p* = 0.046). Laser efficiency, defined as stone volume divided by lasing time, was significantly greater in Group 2 (79.19 vs. 44.51 mm³/min, *p* = 0.031). Intraoperative ventilatory parameters, including respiratory rate, peak airway pressure, PEEP, oxygen saturation, and end-tidal CO₂ levels, were similar between the two groups (all *p* > 0.05). Postoperative outcomes, including residual stone rates, postoperative infection rates, and length of hospital stay, did not differ significantly between the groups (all *p* > 0.05).

**Table 2:** Comparison of perioperative and postoperative parameters

	Group 1 (*n*:42)Standard Tidal Volume	Group 2 (*n*:28)Low Tidal Volume	*P* value
Operation Time (minute)	91 (64-120.75)	93 (64.75–122.5)	0.572
Lasing Time (minute)	30.55 (19.5-40.22)	24.85 (8.77–34.3)	0.056
Lasing Energy (joule)	27.9 (21.85–44.4)	19.08 (9.95–36.1)	**0.046**
Laser Efficiency (mm³/min)	44.51 (25.93–80.43)	79.19 (53.34–96.04)	**0.031**
Respiratory Rate (breaths/min)	12 (12–13)	12 (12–12)	0.136
Peak Pressure (cmH₂O)	17 (15-19.5)	16 (14.75–18.5)	0.505
PEEP (cmH₂O)	5 (5–6)	5 (5–5)	0.157
SPO_2_ (%)	98 (98–99)	99 (98–100)	0.063
ETCO_2_ (mmHg)	33 (31–37)	34 (32–37)	0.698
Residual Stone	6 (14.3)	1 (3.6)	0.145
Postoperative Infection Rate	2 (4.8)	4 (14.3)	0.169
Hospital Stay (day)	1 (1–1)	1 (1–2)	0.525

All continuous variables are presented as median (interquartile range, IQR), and categorical variables are presented as numbers and percentages

*PEEP* Positive End-expiratory Pressure, *SpO₂* Peripheral Oxygen Saturation, *ETCO₂* End-tidal Carbon Dioxide

**Fig. 1:**
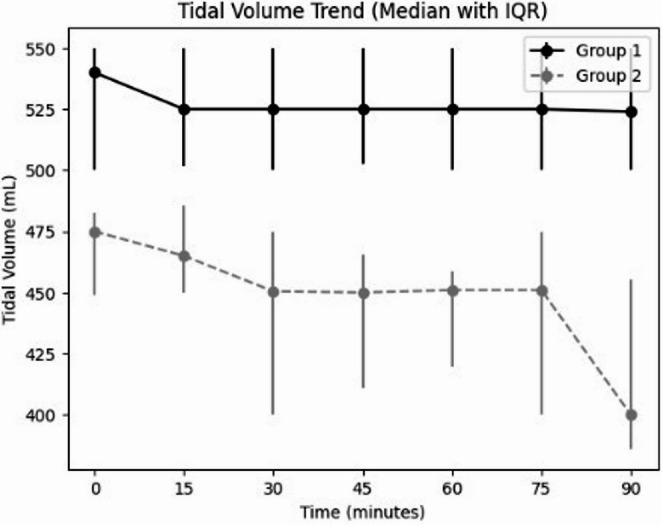
Comparison of tidal volume trends over time between group 1 (≥ 500 mL) and Group 2 (< 500 mL) Data are shown as median (IQR). Group 2 maintained consistently lower tidal volumes throughout the procedure

Across all assessed time points, tidal volume was significantly higher in Group 1 than in Group 2 (all *p* < 0.001) (Fig. 1). The observed reduction in tidal volume over time in the low tidal volume group may be attributed to intraoperative adjustments made by the anaesthesiologist according to patient-specific respiratory parameters and lung mechanics.

## Discussion

In this prospective cohort study, we evaluated the effect of intraoperative tidal volume settings during general anaesthesia on surgical efficiency and outcomes in patients undergoing fURS for renal stones. The findings of the present study partially confirmed our hypothesis. Although lower tidal volume ventilation was associated with improved intraoperative surgical conditions, this did not translate into a statistically significant improvement in stone-free rates. This may be explained by the multifactorial nature of ureteroscopic stone surgery outcomes. Patients were stratified according to a pragmatic tidal volume cutoff of 500 mL based on institutional ventilation practices, and operative parameters, stone-free rates, and complication profiles were compared between the groups. The unequal group sizes and sex distribution differences are likely attributable to the observational non-randomized design of the study. Since ventilation strategies were selected according to routine anaesthetic practice, some degree of selection imbalance may have occurred.

Our results demonstrated that higher tidal volumes were associated with a significant increase in total laser energy usage. Although not statistically significant, the numerically higher stone burden in the higher tidal volume group may have contributed to increased energy usage. This observation should be interpreted cautiously, as differences in stone burden were not statistically significant. In addition, laser efficiency was found to be significantly higher in the group managed with lower tidal volumes. Although stone volume and lasing time did not differ significantly between groups, their ratio was used as an exploratory indicator of procedural efficiency. This derived parameter should be interpreted cautiously and does not represent a clinical outcome. Future studies are required to validate this derived metric in larger and randomized cohorts.

No significant differences were observed between the groups in terms of operative time, stone-free rates, or postoperative complications. Although a trend toward shorter lasing time was observed, this difference was not reflected in total operative time. Although laser efficiency differed between groups, this was not reflected in total operative time, likely because overall procedure duration is determined by multiple operative steps beyond active laser use, many of which are not directly influenced by ventilatory strategy. Moreover, the similarity in respiratory parameters and perioperative safety outcomes suggests that variations in tidal volume within physiological limits do not adversely affect surgical success or patient safety during fURS. Despite lower tidal volumes, adequate oxygenation and ventilation were maintained, as indicated by comparable oxygen saturation and end-tidal CO₂ levels between groups. This suggests that compensatory adjustments in respiratory rate and oxygen delivery were effective in preserving physiological stability. In patients with comparable stone burdens, achieving similar success rates with lower laser energy consumption and higher laser efficiency at lower tidal volumes indicates that a low tidal volume strategy may warrant further investigation to reduce energy usage without compromising surgical efficiency or clinical outcomes.

From a clinical perspective, these findings suggest that intraoperative ventilation strategies may represent a modifiable factor influencing laser energy consumption during fURS. In patients with similar stone burdens, achieving comparable outcomes with lower laser energy consumption and higher laser efficiency at lower tidal volumes suggests that ventilation strategy may represent a modifiable intraoperative factor influencing laser energy usage. A low tidal volume approach may therefore warrant further investigation as a potential strategy to optimize lithotripsy performance without compromising clinical outcomes, particularly in prolonged procedures or patients with larger stone burdens, where minimizing unnecessary energy delivery may theoretically reduce thermal exposure and improve procedural efficiency.

Interest in ventilation-related renal motion during RIRS has increased in recent years, driven by the hypothesis that respiration-induced kidney excursion compromises laser targeting and stone fragmentation efficiency. Kourmpetis et al. introduced the concept of “respiratory-gated RIRS” in a randomized controlled trial, demonstrating that a low-ventilation strategy using reduced tidal volume and respiratory rate significantly improved fragmentation, extraction, and processing rates compared with standard ventilation [9]. Importantly, ventilation mode remained an independent predictor of operative efficiency on multivariable analysis, supporting a mechanistic link between respiratory motion control and surgical performance. Similar to this study, our findings also demonstrated a significant gain in procedural efficiency. In our cohort, laser efficiency was significantly higher in the low tidal volume group, supporting the notion that respiratory modulation can positively influence laser–stone interaction. Reduced respiratory-induced renal motion may allow more stable laser targeting, thereby enhancing effective energy delivery and minimizing unnecessary laser use. These results suggest that, within physiological limits, a low tidal volume strategy may contribute to improved lithotripsy performance, even in settings with experienced surgeons.

Doğan et al. demonstrated that reducing tidal volume while increasing respiratory frequency significantly decreased fluoroscopically measured renal motion during RIRS; however, this did not translate into differences in operative time, stone-free rates, or complication profiles [13]. These findings align closely with our results and suggest that while ventilation strategies can influence renal excursion, their effect on clinically measurable outcomes may be limited in selected patient populations.

Evidence from pediatric cohorts suggests a potentially greater sensitivity to ventilation-related motion. Kılınç et al. reported significantly higher stone-free rates in pediatric patients undergoing RIRS with lower tidal volumes (≤ 7 mL/kg), despite similar operative times and complication rates [14]. Differences in renal size, compliance, and proportional excursion during respiration may partly explain why ventilation modulation appears to have a more pronounced effect in children than in adults.

Alternative respiratory strategies have also been explored. Gadzhiev et al. evaluated a combined respiratory support model using high-frequency jet ventilation with small-volume mechanical ventilation and reported improved surgeon-perceived stability without anaesthesia-related complications [15]. Although largely exploratory, these findings highlight continued efforts to optimize anaesthetic techniques for endourologic precision. The higher total lasing energy observed in the ≥ 500 mL tidal volume group should be interpreted cautiously. Prior literature consistently demonstrates that stone diameter and volume are key determinants of laser energy requirements during lithotripsy, and baseline differences in stone burden may explain this finding rather than a direct effect of ventilation strategy. The absence of corresponding differences in lasing time or operative duration further supports this interpretation.

In light of our findings, modulation of tidal volume within physiological ranges during general anaesthesia does not appear to confer a clinically meaningful advantage in terms of operative efficiency, stone-free rates, or perioperative safety in adult patients undergoing fURS when procedures are performed by experienced endourologists. While higher tidal volumes were associated with increased total laser energy usage, this finding is likely attributable to baseline differences in stone burden rather than a direct effect of ventilation strategy. These results suggest that ventilation-related renal motion control alone may have limited impact on surgical outcomes in routine adult fURS and should be considered an adjunct rather than a primary determinant of procedural success. Further randomized investigations incorporating objective renal motion metrics and a broader spectrum of stone complexity are needed to elucidate the effect of ventilation modulation on endourological efficiency. In addition, subgroup analyses according to age were not performed due to the limited sample size and should be explored in future studies. We believe that future studies with more balanced sex distribution and homogeneous patient populations may help to better define the relationship between ventilatory parameters and surgical outcomes.

## Limitations

This study has several limitations. Group allocation was not randomized and was influenced by anaesthesiologist preference, which resulted in a higher proportion of female patients in the low tidal volume group. This may introduce selection bias. However, the prospective design and predefined ventilation protocols mitigate some of the inherent bias. Moreover, use of a fixed tidal volume cutoff without adjustment for predicted body weight represents a limitation and may have introduced variability in ventilation strategies. In addition, we consider the lack of evaluation of lower pole stones to be a limitation of the study. Another limitation, deep neuromuscular blockade was routinely used in our institutional practice, although it may not be mandatory for all ureteroscopic procedures. Postoperative stone-free status was not evaluated using non-contrast CT in all patients, which may have limited the accuracy of residual fragment detection. Renal motion was not directly quantified, precluding correlation between ventilation parameters and objective kidney excursion. Future randomized trials incorporating direct motion measurement and broader case complexity are needed to better define the clinical role of ventilation modulation during fURS. Finally, intraoperative changes in stone location relative to preoperative imaging were not systematically evaluated, which may represent another limitation.

## Conclusions

The findings of this study suggest a possible association between ventilatory settings and laser efficiency; however, their clinical relevance remains uncertain. Within physiological limits, the use of a low tidal volume strategy was associated with higher laser efficiency, an exploratory metric that should be interpreted cautiously, without compromising operative duration, stone-free rates, or perioperative safety. Therefore, maintaining lower tidal volumes during fURS, while preserving adequate ventilation, may be associated with differences in laser efficiency; however, these findings require validation in randomized studies.

## Data Availability

Data available on request due to privacy/ethical restrictions.

## Declarations

### Permission to reproduce material from other sources

Not available.

### Clinical trial registry

ClinicalTrials.gov.

### Clinical trial number

NCT07304297.

### Date of registration

12-12-2025.
